# Evidence-Based Toxicology—Hypothesis Testing in Randomized Clinical Trials: Part II—Equivalence

**DOI:** 10.1007/s13181-025-01110-8

**Published:** 2026-01-09

**Authors:** Joshua Trebach, Ali Graebner, Mark K. Su

**Affiliations:** 1 Department of Emergency Medicine, Division of Medical Toxicology, University of Iowa Hospitals and Clinics, Iowa City, IA USA; 2 Iowa Poison Control Center, Sioux City, IA USA; 3 New York-Presbyterian Brooklyn Methodist Hospital, Brooklyn, NY USA; 4 Ronald O. Perelman Department of Emergency Medicine, Division of Medical Toxicology, NYU Grossman School of Medicine, New York, NY USA; 5 Department of Health and Mental Hygiene, New York City Poison Control Center, New York, NY USA

**Keywords:** Equivalence trial, Clinical trial, Randomized clinical trial

“Two things can be true at once.”

-Lara Heacock

Randomized clinical trials with the equivalence design attempt to determine if two treatments or interventions produce similar outcomes within a predetermined margin. For example, agencies such as the Food & Drug Administration may require equivalence trials (specifically, “bioequivalence”) to show that a new generic drug performs similarly to a brand name drug in terms of pharmacokinetics [1]. Equivalence trials can also be used to make assessments about different treatment regimens [2]. Essentially, equivalence trials are necessary to determine when two interventions have a similar beneficial outcome.

While randomized controlled trials in medical toxicology are relatively uncommon, well-designed equivalence trials can be impactful. Equivalence trials test a hypothesis (assumption) that two treatments are equivalent to each other. For example, if a researcher wants to perform an equivalence trial between an equivalence trial between a hypothetical new drug (WAC) compared to N-acetylcysteine (NAC) for the treatment of acetaminophen toxicity, the equivalence trial would be the correct study design. This equivalence trial could then be set up as follows:

- NAC: N-Acetylcysteine (standard, existing drug).
- WAC: Hypothetical new drug.
- Null Hypothesis (H_0_) = WAC is not equivalent to NAC in the treatment of acetaminophen toxicity.
- Alternative Hypothesis (H_a_) = WAC is equivalent to NAC in the treatment of acetaminophen toxicity.

In an equivalence trial, the alternative hypothesis is the new treatment is *equivalent* to the standard treatment.

A common misconception regarding equivalence trials is that when the null hypothesis is not rejected, the new drug (i.e., WAC) is better or worse than NAC. However, an equivalence trial only addresses whether or not the new drug is equivalent to the standard drug, not if the new drug is better or worse than the standard drug.

Before conducting the equivalence trial of WAC compared to NAC, we must define the equivalence margin (δ), (i.e., the acceptable range of difference in outcomes for the two treatments to be considered equivalent). The equivalence margin in this context typically represents the largest, clinically acceptable difference between the two treatments that still allows the treatments to be considered “equivalent.” In this hypothetical example of WAC compared to NAC, we will conclude that WAC is equivalent to NAC if it performs within the effect size margins (i.e., zone of indifference). In our hypothetical equivalence trial of WAC compared to NAC, if the primary outcome is hepatotoxicity (defined as an AST > 1000), the effect size margins would be represented by the percentage of patients that developed hepatotoxicity in patients treated with WAC compared to NAC. In equivalence trials, t is important to choose accurate, measurable, and feasible primary outcomes when attempting to determine equivalence between two treatments.

**Fig. 1:**
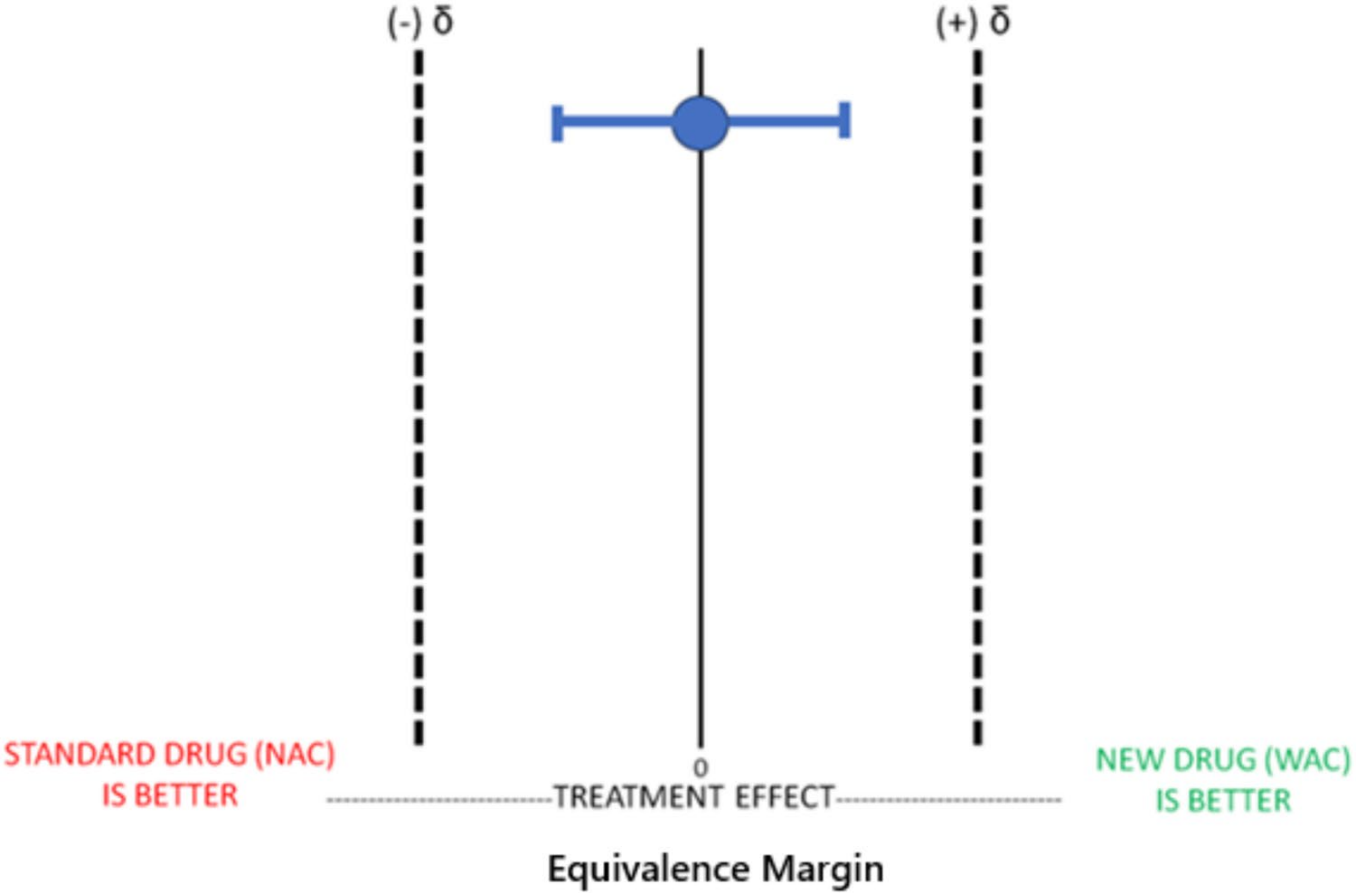
Equivalence Trial: WAC Equivalent to NAC. Legend: In an Equivalence Trial, for the new experimental drug WAC to be considered equivalent to the gold standard treatment NAC, the mean treatment effect and its CI (typically 95%) must fall between the a priori estimated effect size difference (δ), or zone of indifference. In this example, WAC demonstrates equivalence to NAC because it meets this definition

This figure above (Fig. 1) represents that WAC is equivalent to NAC as our mean value and our confidence intervals are within our equivalence margins.

**Fig. 2:**
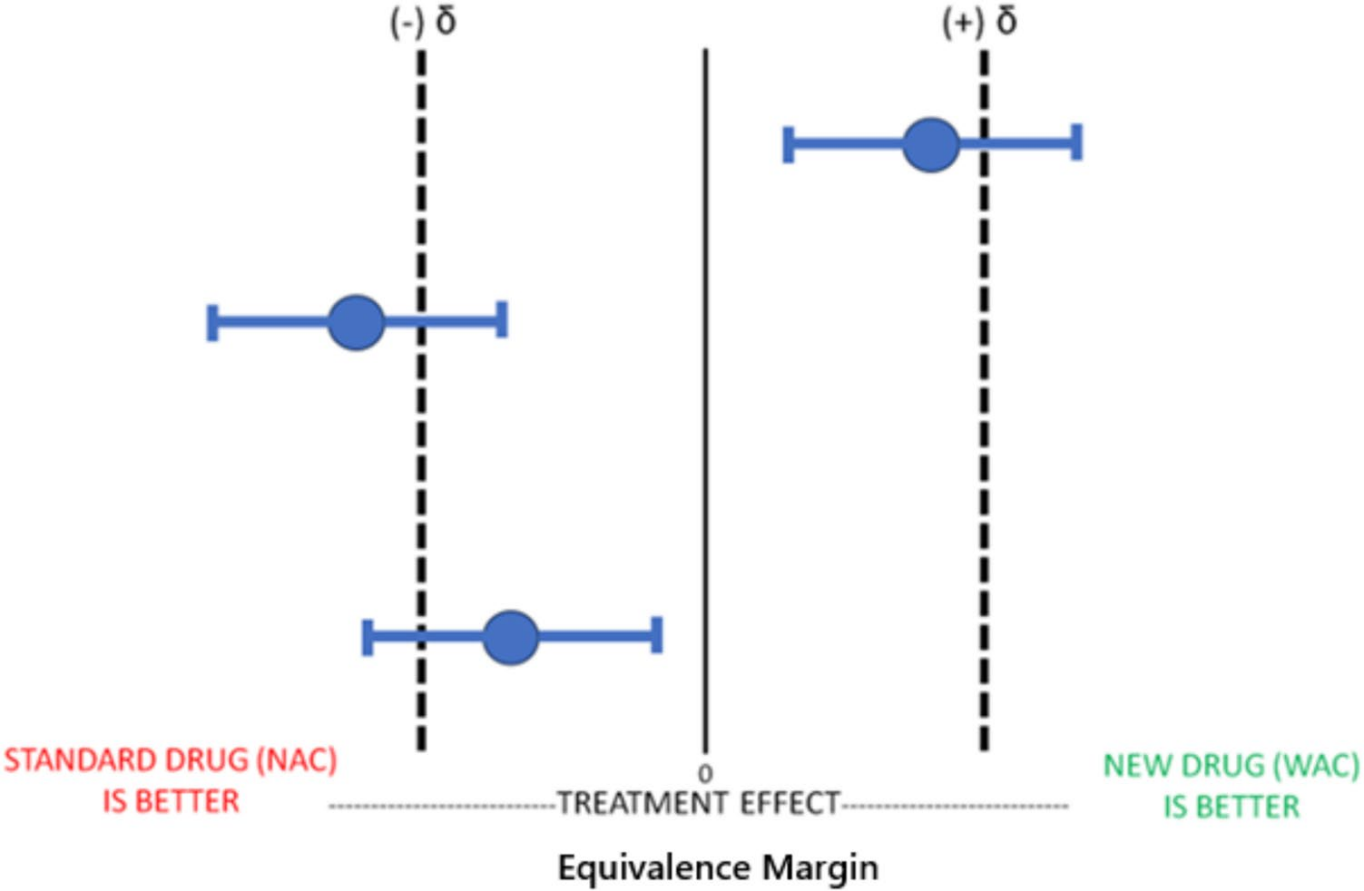
Equivalence Trial: WAC Not Equivalent to NAC. Legend: This figure illustrates the results of three separate equivalence trials comparing the experimental drug WAC to the gold standard therapy NAC. In Equivalence Trials, the new experimental drug WAC cannot be considered equivalent to the standard treatment NAC in any of these three trials because the mean treatment effects and their CIs are either overlapping with the a priori estimated effect size difference/zone of indifference (δ). All three trials would therefore be considered “not equivalent”

The figure above (Fig. 2) represents multiple iterations where WAC is not equivalent to NAC as our mean value and/or their corresponding confidence intervals are not within our equivalence margins. Once again, it should be noted that if the trial does not find WAC to be equivalent to NAC, then WAC can be either better or worse than NAC.

An equivalence trial that demonstrates equivalence simply means that the two treatments performed within the predetermined margin of equivalence. Just because two treatments are equivalent does not mean they have equivalent mechanisms of action, structures, safety profiles, and so forth.

Equivalence trials have an important role and are useful to compare one intervention to another in order to determine if they have a similar beneficial outcome. However, as stated above, failure to prove equivalence does not demonstrate if something is either better or worse. In fact, finding that two treatments are equivalent could simply demonstrate they are equally harmful. Furthermore, finding that two treatments are equivalent does not mean that the new treatment should be immediately adopted or that the two treatments are interchangeable. For example, in an equivalence trial of oral colchicine vs prednisone treatment for acute calcium pyrophosphate crystal arthritis, with a primary endpoint of change in joint pain at 24 h measured by visual analogue scale, equivalence was found between the two drugs for treatment of joint pain. However, despite the finding of equivalence, the authors still advocated that choosing between the two drugs should be guided by comorbid conditions due to potential concerns for adverse effects associated with colchicine [3]. In other cases, even when equivalence is demonstrated between two drugs, factors such as cost or convenience can influence the decision to use the new treatment.

A particular challenge with equivalence trials is how equivalence margins are determined. Previously published papers can be extremely helpful in laying down the foundation for new research. However, it is essential to determine whether previously published data were acquired with appropriate rigor, and that these data have external validity. If there are no previously published data, or rigorous existing studies, experts, or groups of experts, can be cited from narrative or systematic reviews but these may not be ideal either. One can see how this may prove challenging. In the end, the investigators must do their best with all the available existing evidence to determine a reasonable approach to defining equivalence margins in these trials. If these margins “make sense” and a new trial shows that two treatments are equivalent, these findings can be potentially tremendous and “practice-changing.”

An additional challenge with equivalence trials is that they typically require larger sample sizes than superiority trials. While superiority trials aim to detect a difference between two treatments (i.e., is one superior to another?), equivalence trials have to rule out both inferiority and superiority—in other words, superiority trials are a one-sided test whereas equivalence trials are a two-sided test. Due to the nature of being a two-sided test, in equivalence trials, the sample size needs to be sufficiently high so that an appropriate level of power is achieved.
